# Optimization of microwave-assisted biodiesel production from watermelon seeds oil using thermally modified kwale anthill mud as base catalyst

**DOI:** 10.1016/j.heliyon.2023.e17762

**Published:** 2023-07-01

**Authors:** Sunday Chukwuka Iweka, Olayomi Abiodun Falowo, Adebimpe Amos Amosun, Eriola Betiku

**Affiliations:** a Department of Mechanical Engineering, Delta State University of Science and Technology, Ozoro, Nigeria; b Department of Chemical Engineering, Landmark University, Omu-Aran, Nigeria; c Center for Energy Research and Development, Obafemi Awolowo University, IIe-Ife, Nigeria; d Department of Biological Sciences, Florida Agricultural and Mechanical University, Tallahassee, FL, 32307, United States

**Keywords:** Machine learning, Biowaste catalyst, Optimization, Watermelon seed oil, Microwave-assisted transesterification, Biodiesel

## Abstract

A heterogeneous catalyst was developed from raw Kwale red Anthill mud by thermal treatment in a muffle furnace at 900 °C for 4 h. The resulting heterogeneous catalyst was highly porous with a surface area of 42.16m^2^/g, possessing excellent stability as well as high catalytic activity. Central Composite Design and Machine Learning approach (Python code) were applied to model and optimize biodiesel yield from extracted watermelon oilseed. Highest biodiesel yield of 93.41 wt% was obtained under the experimental conditions of 4min duration, 350 W microwave power, 4 wt% of catalyst, and MeOH/oil ratio of 8:1 based on Central Composite Design rotatable. The optimum value of the biodiesel yield from Machine Learning was 91.7 wt%, showing a marginal performance over the Central Composite Design rotatable value (91.6 wt%) at the optimized conditions of 3 min, 280 W, 3 wt% catalyst loading and MeOH/oil molar ratio of 6:1. The correlation of the coefficient (R^2^) of the model was 0.9827 for Central Composite Design rotatable while the R^2^ of the Machine Learning model was 1.0. Thus, python coding in terms of prediction and accuracy of biodiesel yield was superior to Central Composite Design rotatable, even though both models provide a reliable response within the region of data analyzed. The Gas Chromatography-Mass Spectroscopy of the biodiesel produced revealed the presence of both saturated and unsaturated fatty acid methyl esters. Biodiesel properties from watermelon seed oil transesterification fall within the recommended standard for biodiesel fuel. This study concluded that an effective green biowaste catalyst generated from earthen waste could enhance biodiesel production from watermelon seed oil, hence, ensuring sustainability and economic feasibility for biodiesel industries.

## Graphical abstract

**Figure:**
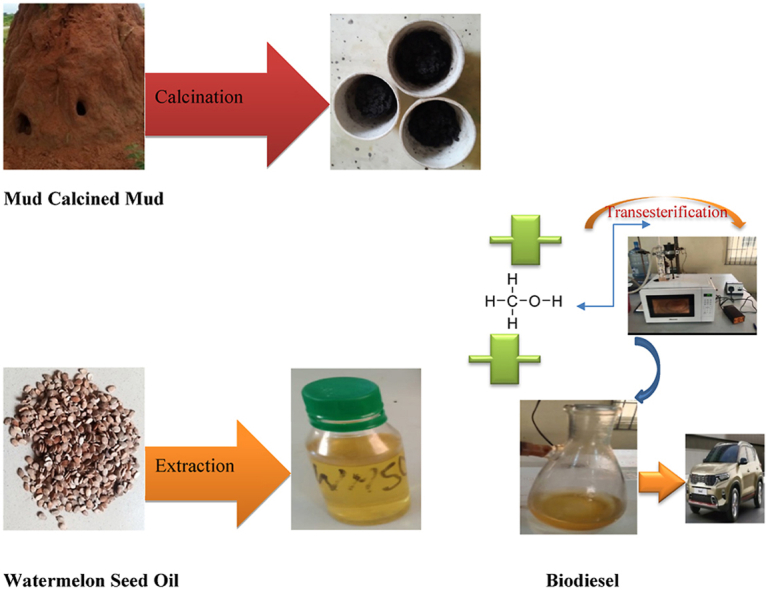

## 1 Introduction

Nearly all human activities are driven by energy or application of energy in one form or another. Development at local and global levels as well as the industrialization of our world are made possible by energy. There exist global challenges to the usage of fossil fuels such as the rising cost of crude oil, price fluctuation on the international stage, the generation of environmental pollutants, and rising energy demand due to the increase in population. A need for renewable energy that reduces the rate of environmental pollution due to greenhouse gas emissions, and over-dependence on the non-renewable source of energy [1] is desirable. Currently, fossil fuel is fast depleting coupled with the environmental threat resulting from its usage; global warming due to the emission of mineral fuels combustion has made renewable energy more secure for the future of the environment [2]. Implementation and sustainability of renewable energy at different levels or countries would reduce the overdependence on existing finite fossil oil which is sometimes used as an economic threat. Renewable energy resources are being explored, and some of these technologies are currently at the advanced stage, resources such as water, biogas, biodiesel, wind, solar, geothermal, and nuclear energy [3] are contributing a considerable share to the energy mix. Solar and wind energy are less appealing due to the global weather (seasonal variation), and geothermal energy is not ideal due to the destruction of the earth’s crust. Utilizing nuclear energy may also cause radioactive leakage. Additionally, biogas production takes time due to full anaerobic digestion and its complex production system, in contrast to biodiesel, which is simple to produce, takes less time and has readily available materials. Water is also scarce in desert regions, which restricts its use in energy generation. The ease at which biodiesel can be turned into liquid fuel for use as a transportation fuel is another characteristic that sets it apart from other renewable energy sources [4]. As a result, the cost of biodiesel is lower, and it produces a greener form of energy [5]. Biodiesel is capable of partially substituting diesel fuel produced from fossil oil because biodiesel has a higher oxygen content than diesel fuel and a higher flash point. Thus, it is preferable to diesel fuel [[5], [6], [7]]. Biodiesel, known as green diesel is derived from oleochemical feedstocks in plants and animals. Moreover, it is renewable, biodegradable, portable, and environmentally friendly since it emits low aromatic and sulfur compounds [3,8]. Biodiesel can be categorized based on the feedstock used in its production; edible, inedible, used cooking oil, and microalgae are categorized as 1st, 2nd, 3rd, and 4th generation feedstocks, respectively [3,9,10]. Biodiesel produced from non-edible feedstocks is very attractive globally due to its renewability, cleanliness, sustainability, lubricity, eco-friendliness, and non-competitiveness with foods [11,12].The long chains of biodiesel fuel are generated through the transesterification process of triglycerides using methanol with (or without) a catalyst [13,14].

Technologies used in the production of biodiesel include transesterification, thermal cracking (pyrolysis), micro-emulsions, direct use and blending of vegetable oils, and pyrolysis [15]. Transesterification, which is the most popular technology today converts vegetable oil and short alcohol (C1–C2) to biodiesel in the presence of a base catalyst. Transesterification reaction uses any of these three conversion heating methods: supercritical heating, conventional heating, and microwave heating [16]. Overall, in terms of effectiveness and eco-friendliness, microwave-assisted transesterification is the most practical of the three technologies [3]. Microwave-assisted transesterification can generate cleaner reaction products within a short reaction time due to its rapid heating. In addition, benefits of the microwave-assisted transesterification technique include shorter reaction time, less solvent use, a higher biodiesel yield, and less energy use [17,18]. Thus, microwave devices can trigger transesterification even in the absence of a solvent, providing a green chemistry solution to a variety of environmental issues involving dangerous and toxic contaminants [16].

Homogeneous catalysts continue to be the catalyst of choice for large-scale biodiesel production due to their availability, inexpensive cost, and ability to catalyze at moderate operating conditions [19]. However, the continual usage of these catalysts is not favorable to the biodiesel economy and the environment. In light of these demerits, heterogeneous catalysts have advantages over homogeneous catalysts and can make biodiesel production sustainable and profitable. Solid catalysts can be easily recovered and reused severally without any threat to resources and the environment. A safer and green approach is to develop heterogeneous catalysts for biodiesel production from agricultural wastes or inexpensive abundant resources. Recently, earth crusts such as boulders, clays, and termite hills were considered as catalyst precursors due to their efficiency, durability, availability, and efficacy. A termite hill is a mass of sand, clay, or a mixture of clay and other materials that accumulate at the entrances to ant colonies' underground homes [20]. The earth’s crust is found to be an aluminosilicate substance with a wide range of chemical and physical diversity. While Si, Al, Ca, Mg, K, Ti, and Na are among the most frequent elements found in an earth’s crust, the chemical and mineralogical makeup of the termite hills varies depending on the region. A potassium fluoride-treated clay catalyst converts soybean oil to 99.7 wt% biodiesel under 1 h and methanol/oil molar ratio 6:1 [21].

To ensure that the biodiesel economy is appealing, the biodiesel industry must address food issues and kick-start economic development by switching from using edible seed oils like palm, soybean, and coconut to non-edible seed oils like *jatropha curcas*, rubber seeds [5], and watermelon seeds. Modeling biodiesel yield, as well as process inputs, would make biodiesel production economically viable. RSM consists of many packages, but CCD outperforms the Box-Behnken design (BBD) because it predicts values that are more accurate to the actual response and is more robust [22]. Machine learning is a pre-programmed method that utilizes successive iterations techniques using extrinsic data serving as training input to stepwise update problem-solving abilities, and self-mastery to answer the challenging issues [23,24]. Due to its brain’s capacity for auto-learning and self-improving, machine learning (ML), a subset of artificial intelligence (AI), is crucial in the designing, operating, controlling, optimizing, and monitoring of systems [24]. Powerful machine learning algorithms are used in various ML techniques, including Decision Trees (DT), Linear Regression, Principal Component Analysis (PCA), Artificial Neural Networks, and Genetic Algorithms (GA) [23]. In comparison to other modeling techniques like RSM, Genetic Algorithm, and Taguchi method, it has been shown that machine learning offers higher forecasts and accuracy during the production of biodiesel. According to Ref. [25] ANN capability was compared with CCD model in the transesterification of castor oil and methanol in the presence of H_2_SO_4_, it was reported that ANN performed better than CCD. Likewise, a *jatropha*-algae oil blend transesterification study conducted by Ref. [26] reported that ANN performed better than RSM. Regression, artificial neural network (ANN), and analytical methods are currently the most popular modeling techniques for biodiesel research [[27], [28], [29]].

The biodiesel produced from the watermelon seeds in previous studies applied Sodium hydroxide [30], and potassium hydroxide as a base catalyst, and the transesterification carried out using a hotplate with magnetic stirrer [31,32]. In another study, ultrasonication and microwave-assisted transesterification of watermelon seed oil were performed using crystalline Mn (II) carbonate as a catalyst [33]. The challenges associated with the use of mineral catalysts and conventional heating remain a matter of concern. To have a cost-effective biodiesel production process, a heterogeneous catalyst from a renewable source was applied to investigate biodiesel production from watermelon seed oil (WMSO) in a process intensification device. In this study, a green heterogeneous amphoteric-base catalyst developed from Kwale Red Anthill mud was used to generate fatty acid methyl ester from extracted watermelon seeds oil. Biodiesel production in the microwave-assisted transesterification of WMSO was modeled using CCD and the linear regression model of ML.

## 2 Materials and methods

### 2.1 Collection of materials

The watermelon seeds were gathered from a restaurant at the Delta State University of Science and Technology in Ozoro, Nigeria. Red Anthill mud was gathered from a farm along the Kwale-Ogwash road, Kwale, Nigeria (5°42′ 27″ N). The chemicals and reagents used for the oil physicochemical determination and biodiesel characterization are all analytical grade.

### 2.2 Extraction of the watermelon seed oil

**Fig. 1:**
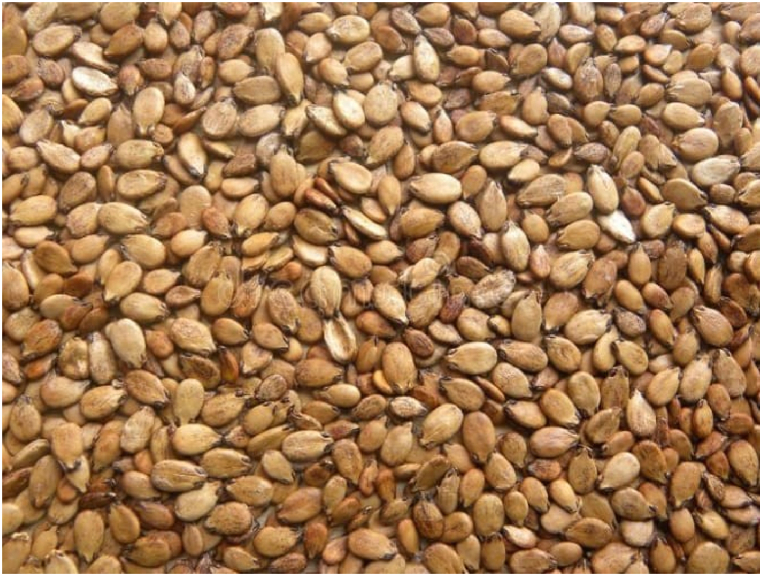
Watermelon seeds.

The watermelon seed oil utilized for biodiesel synthesis was extracted at the Petrochemical Technology laboratory, Afe-Babalola University, Ado-Ekiti. Fresh oil was obtained from pulverized oilseeds using a Soxhlet-unit method as *n*-hexane acts as the solvent of extraction. Typically, watermelon seeds were washed severally to remove dirt using tap-distilled water. The sample after washing was sun-dried until a steady weight was achieved. Dried watermelon seeds used in this study are displayed in Fig. 1. A hand-powered bench grinder was used to mill the seeds after they had been separated from the kernel. In the thimble of a Soxhlet extractor, a measured volume of *n*-hexane was introduced to solubilize a given mass of pulverized watermelon seeds. A condenser was firmly attached to the other end of the extractor unit to prevent solvent loss, a round-bottom flask containing the extraction solvent was linked to the extractor, and the hotplate was turned on. After the completion of the extraction process, fresh oil was recovered from the solvent at 65 °C. Thereafter, the oil was dried and quantified gravimetrically according to Equation (1).

(1) $$Oil\;yield\;\left(wt.\%\right)=\frac{W_{o}}{W_{S}}\times 100$$

where W_o_ represents the weight of the extracted WMSO (g) while W_S_ represents the weight of the watermelon seed used for extraction (g).

The physicochemical properties (acid value, moisture content, pH, density, kinematic viscosity) of the extracted WMSO was measured using standard methods [34].

Acid value is the amount of potassium hydroxide, measured in milligrams, required to neutralize the free fatty acid present in 1 g of oil. The titration method was used to determine the acid value of WMSO. A known mass of WMSO (g) was measured and equal volume of diethyl ether and ethanol were added to the measured oil. Two drops of phenolphthalein indicator were added to the oil solvent mixture, and the solution was titrated against 0.1 M KOH in burette. The acid value of WMSO was obtained using Equation (2).

(2) $$Acid\;Value\;\left(A.V\right)\;in\;mg\;KOH/g=\frac{56.1\;x\;V\;x\;M}{W}$$

where: V = volume of KOH used, M = Molarity of KOH, W = Weight of oil.

### 2.3 Preparation of KRAM catalyst

**Fig. 2:**
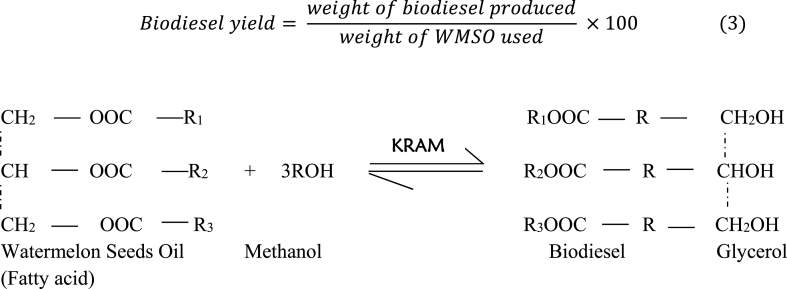
The transesterification reaction of WMSO biodiesel.

The Kwale Red Anthill Mud (KRAM) used for catalyst synthesis was collected from Kwale-Ogwash road, Kwale. This catalyst precursor (Fig. 2) was an abandoned earth soil rich in minerals. The harvested red anthill mud was first sifted to remove dirt and stones. The anthill mud was broken up into little pieces, washed with distilled water, and dried at 120°C for 4 h. Using a mortar and pestle, the dried red anthill mud was grounded into a fine powder, and was sieved to sizes ranging between 150 and 220 mm. Then, the calcination of resulting fine particles was initiated at 900°C for 4 h using an electric furnace at a heating rate of 10°C/min [19,21]. The heat-treated ash of red anthill mud after cooling was crushed into a fine powder and sieved. The calcined KRAM sample was kept in a screw-top bottle and used for further analysis as well as biodiesel production.

### 2.4 Characterization of Kwale Red Anthill Mud catalyst (KRAM)

Characterization of synthesized catalyst (KRAM) was carried out to investigate the catalytic potential of the carbonized specimen. The surface morphology and mineral composition of KRAM were examined using a high energy-dispersive X-ray analyzer linked to a scanning electron microscope (SEM, Hitachi SU 3500 scanning microscope, Tokyo, Japan). Using an X-Ray Diffractometer, the phases and crystal structures of the carbonized sample were studied (Shimadzu XDS 2400H). The diffraction pattern was recorded in the 2θ range of 10–80 with a variation of ±0.05°, and at a scan speed of 2 °C/minute for 120 min. The surface characteristics of the synthesized catalyst were studied using the N_2_ adsorption-desorption method. Before analysis, the samples were degassed using the micromeritics flow prep 067 in combination with nitrogen gas for 3 h at a temperature of 473 K. The degassed sample was reweighed, the analysis was completed in the liquid nitrogen-conditioned micromeritics Tristar 3000 V4.02, and the results were recorded. The catalyst surface area, total pore volume, and pore size of the calcined sample were obtained using N_2_ an isotherm [35]. The composition of the KRAM sample was examined using an X-ray fluorescence analyzer (Shimadzu EDXRF-702HS). All measurements were taken from the instrument operating at 40 kV and 18 mA. The collimator of choice was 10 mm, with a 100-s counting period. The Shimadzu EDX software program was used to extract the number of counts per second (cps/μA) for element Kα from the sample X-ray spectrum.

### 2.5 Transesterification of watermelon oil

The esterification of extracted oil was performed since the watermelon oil has an acid value of 9.16 mg KOH/g. Esterification of WMSO was performed at the following conditions; molar MeOH/WMSO of 7:1, H_2_SO_4_ concentration of 1 wt% for 2 h at a temperature of 60 °C. A 500 mL two-neck glass reactor was used for the transesterification of the esterified WMSO in a modified microwave (model: H20MOW Hisense). To the modified device, a reflux condenser system was attached to the glass reactor to prevent evaporation of the reaction mixture, and an external stirrer having two blades controlled by a 12v electric motor (12v HUPE DC Stabilizer) was also attached. A CCRD design was used to model WMSO transesterification. The transesterification of WMSO was carried out according to the stipulated conditions in the experimental design. After the finalization of the transesterification, the resultant biodiesel-glycerol mixture was subjected to centrifugation at 8000 rpm for 7 min. The product mixture containing biodiesel was washed with warm distilled water three times. Equation (3) was used to compute the biodiesel yield. Fig. 2 illustrates the transesterification reaction of watermelon seeds oil in the presence of catalyst (KRAM).

(3) $$Biodiesel\;yield=\frac{weight\;of\;biodiesel\;produced}{weight\;of\;WMSO\;used}\times 100$$

### 2.6 Biodiesel physical and chemical properties

Biodiesel synthesized from the transesterification of WMSO was tested to ascertain its purity and quality. Standard techniques were used to determine the biodiesel’s physical and chemical characteristics, including its specific gravity, acid value, kinematic viscosity, density, caloric value, and cetane number [34].

## 3 Results and discussion

### 3.1 Physiochemical properties of the Extracted oil from watermelon seed

The physicochemical properties of the extracted oil from watermelon seed are presented in Table 1. The oil yield (47.61 wt%) from the watermelon seeds is similar to oil recovery from *Sesamum indicum* seeds (48%) [36]. The acid value of the oil which is an index of free fatty acid content was low, however, transesterification of the extracted oil would require a pretreatment step to be carried out. The WMSO possessed excellent properties which makes it a potential feedstock for biodiesel production. Properties such as kinematic viscosity, density, pour point, cloud point, and pH indicate that the extracted oil could be easily converted to biofuel. The saponification value of WMSO was less than 200 mgKOH/g which indicates that the extracted oil has a low tendency to form soap during the transesterification process. The peroxide value of the oil is less than 10 mg/g which depicts that the oil has high resistance to peroxidation during storage. The iodine value of WMSO is high, indicating that the oil is semi-dry oil.

**Table 1:** Physiochemical properties of watermelon seed oil.

Properties	Value
Yield (wt%)	47.61
pH	7.3
Moisture content (%)	4.60
Density (g/cm^3^	0.9407
Kinematic viscosity (mm^2^/s) at 40 °C	1.35
Cloud point (°C)	9.0
Pour point (°C)	5.0
Acid value (%)	9.16
Free fatty acid (%)	4.58
Peroxide value (mg/g)	8.53
Iodine value (mgI_2_/g)	142.50
Saponification value (mgKOH/g)	186.24

### 3.2 Results of characterization of calcined Kwale Red Anthill Mud (KRAM)

#### 3.2.1 Brunauer-Emmet-Teller (BET) surface area

The surface area, pore volume, porosity, and pore diameter of calcined Kwale Red Anthill mud were ascertained using the N_2_ adsorption-desorption technique as presented in Table 2. The surface area and pore volume in this study are comparable to the value obtained in Ref. [37] for bentonite clay which is 60 m^2^/g and 0.0294 cm^3^/g respectively.

**Table 2:** Surface area properties of KRAM.

Parameter	Value
Surface area (m^2^ g^−1^)	42.16
Pore volume (cm^3^ g^−1^)	0.029
Pore Diameter (nm)	0.79
Porosity (%)	25.10

#### 3.2.2 EDX result of calcined KRAM

**Fig. 3:**
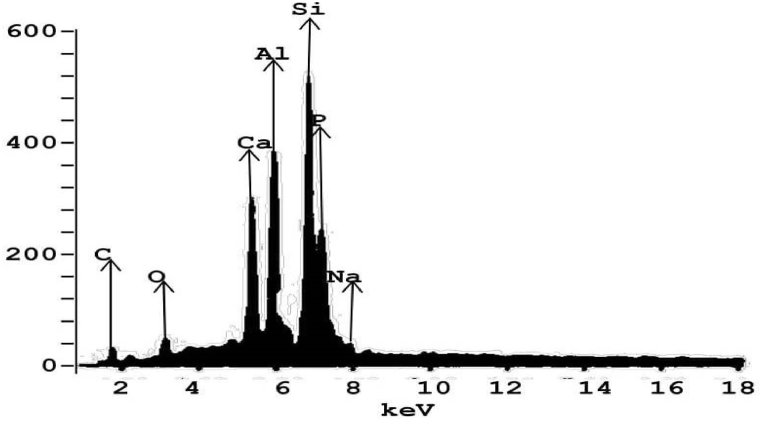
EDX plot of KRAM catalyst.

The EDX spectrum of KRAM is shown in Fig. 3. Chemical analysis of the synthesized catalyst shows that KRAM catalyst was dominated by Silicon, Aluminum, Calcium, etc. Table 3 shows that the group 1 element (Na) was present in very small amounts. The main minerals in the sample are silicon, aluminum, and calcium as they formed the majority of the KRAM catalyst composition. Similar findings have also been reported for termite hill catalyst [20], and natural clay [38,39]. Silicon a metalloid has the largest weight fraction, which is indicative of the catalyst precursor. The main metals (Al and Ca) in the catalyst can readily give electrons to other non-metals. And these metals in addition to Silicon (Si), a metalloid makes ideal catalysts.

**Table 3:** Elemental composition of calcined Kwale red Anthill Mud.

Element	Composition (%)
Silicon (Si)	46.90
Carbon (C)	2.56
Oxygen (O)	4.91
Calcium (Ca)	14.08
Sodium (Na)	0.65
Aluminum (Al)	21.04
Phosphorus (P)	9.82

#### 3.2.3 XRD results

**Fig. 4:**
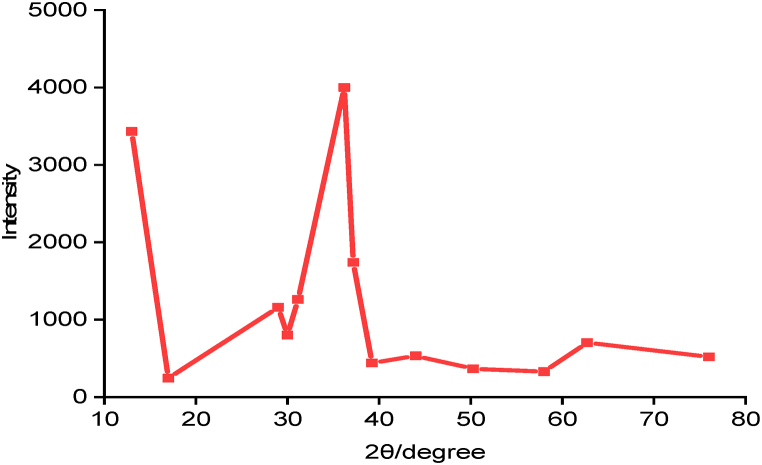
XRD pattern of KRAM.

The crystalline phases of the synthesized catalyst are revealed using XRD analysis (Table 4). The XRD pattern indicated that three crystal structures were present, and the phase is dominated by Kaolinite. There are multiple sharp diffractions in the calcined KRAM, notably peaks at 2θ = 13.00°, 30.00°, 36.21°, and 44.00° indicates the existence of kaolinite. The peaks at 2θ = 17.00°, 39.22°, and 58.00° are assigned to goethite structure, whereas the peaks at 2θ = 28.98°, 31.15°, 37.18°, 50.26°, 62.71°, and 76.00° are attributed to quartz structure (Shimadzu XDS 2400H). The XRD measurement and the EDX result both support the successful heat dispersion on the red anthill mud. The diffraction of XRD using Origin Software is depicted in Fig. 4.

**Table 4:** Diffraction results of KRAM Catalyst.

2θ/degree	Plane	Intensity	d-Valve (Å)	Minerals	% Composition
13.00	0 0 1	3432.82	6.8101	Kaolinite	20.42
17.00	0 0 2	245.31	5.2158	Goethite	7.19
28.98	1 1 2	1158.52	3.0811	Quartz	8.39
30.00	1 1 0	800.09	2.9787	Kaolinite	4.86
31.15	2 1 0	1260.42	2.8711	Quartz	7.51
36.21	2 3 1	4000.75	2.4808	Kaolinite	24.95
37.18	1 1 3	1740.88	2.4185	Quartz	10.20
39.22	1 2 4	439.5	2.2971	Goethite	2.70
44.00	1 4 4	532.61	2.0579	Kaolinite	3.30
50.26	2 0 2	365.67	1.8152	Quartz	2.11
58.00	4 0 0	330.26	1.5901	Goethite	1.71
62.71	0 0 4	702.73	1.4472	Quartz	3.90
76.00	3 1 1	519.65	1.2522	Quartz	2.74

#### 3.2.4 Results of XRF analysis

XRF measurement of calcined red anthill mud shows the mineralogical components of the sample. Silica (SiO_2_) and alumina (Al_2_O_3_) are two of the sample’s most prevalent oxides, according to Table 5. This result having SiO_2_ and Al_2_O_3_ as the major oxides are consistent with the chemical analysis of calcined termite hills and are close to the % composition reported by Ref. [20]. Due to the presence of amphoteric oxides, the synthesized KRAM could be assumed to have a dual-active site. The synthesized catalyst also contained strong basic oxides (MgO, Na_2_O, K_2_O, and CaO) with a total of 7.94 wt%, the presence of different oxides on the catalyst surface would provide adequate catalytic sites for triglycerides-methanol during transesterification [40].

**Table 5:** Elemental composition of KRAM from XRF analysis.

S/n	Basic Oxides	Formulae	% Composition
1	Silicon Oxide	SiO_2_	63.48
2	Aluminum Oxide	Al_2_O_3_	19.00
3	Calcium Oxide	CaO	0.06
4	Iron Oxide	Fe_2_O_3_	3.49
5	Magnesium oxide	MgO	4.43
6	Potassium Oxide	K_2_O	1.62
7	Chromium Oxide	Cr_2_O_3_	0.03
8	Sodium Oxide	Na_2_O	1.83
9	Phosphorus Oxide	P_2_O_5_	4.35
10	Sulfur trioxide	SO_3_	0.08
11	Manganese Oxide	MnO	0.05
12	Titanium Oxide	TiO_2_	0.16
13	Zinc Oxide	ZnO	0.03
14	Copper Oxide	CuO	0.01
15	Loss of Ignition	LOI	1.27

### 3.3 Modeling results of WMSO transesterification

Biodiesel from watermelon seed oil and methanol in the presence of calcined Kwale Red Anthill Mud was modeled using a rotatable design of CCD. The irradiation time, irradiation power, catalyst loading, and MeOH/Oil ratio are the four design variables modeled to investigate their effects on the biodiesel yield. The CCD model generated thirty (30) experimental runs, and the response of each run was presented in Table 6 in a microwave-assisted transesterification process. The highest biodiesel yield (93.41 wt%) was observed at the following process conditions; time of 4 min, 350 W irradiation power, catalyst amount of 4 wt%, and MeOH-oil ratio of 8:1. On the contrary, the lowest biodiesel yield (86.38 wt%) from WMSO transesterification was obtained in run 13 at the following conditions; time of 4 min, irradiation power of 210 W, catalyst amount of 4 wt%, and MeOH-oil molar ratio of 4:1. The effect of the irradiation power and methanol to oil molar ratio seems to be the major driver of this transesterification process.

**Table 6:** Biodiesel yield from CCD rotatable modeling of WMSO transesterification.

Run	A: Time (mins)	B: Irradiation power (W)	C: Catalyst amount (wt.%)	D: MeoH:Oil molar ratio	Biodiesel yield (wt.%)
1	3	280	3	10	89.54
2	2	350	4	8	89.22
3	3	280	3	6	91.60
4	3	280	3	2	89.74
5	4	350	2	8	89.08
6	4	350	4	8	93.41
7	3	140	3	6	89.72
8	4	350	4	4	90.64
9	5	280	3	6	89.99
10	3	280	3	6	90.94
11	2	210	2	8	89.08
12	2	350	2	4	90.18
13	4	210	4	4	86.38
14	3	420	3	6	92.3
15	1	280	3	6	88.3
16	4	350	2	4	93.28
17	2	210	4	8	90.58
18	3	280	3	6	90.32
19	2	210	4	4	87.57
20	3	280	5	6	87.7
21	2	350	2	8	86.83
22	3	280	3	6	90.87
23	2	350	4	4	87.5
24	4	210	2	8	86.74
25	3	280	1	6	88.64
26	2	210	2	4	92.21
27	4	210	2	4	90.13
28	3	280	3	6	91.13
29	3	280	3	6	90.93
30	4	210	4	8	89.63

The significance of the process conditions investigated in this study and the degree of influence on biodiesel yield within the region considered was examined using Analysis of Variance (ANOVA). ANOVA obtained after regression analysis of the biodiesel yields from WMSO using the calcined KRAM was depicted in Table 7. F-value of 60.72 and a p-value ≤0.0001 indicate that the transesterification process model is significant. Any term having a p-value ≤0.05 is adjudged to be a significant term [6,41]. The process factors such as time, irradiation power, MeOH-oil molar ratio, and catalyst amount are all significant. Meanwhile, some interactive terms such as AD and quadratic term B^2^ are insignificant, because their values are greater than 0.1. The model’s lack of fit is insignificant relative to pure error and the lack of fit has an F-value of 0.49 which imply that 84.54% possibility of being caused by the noise.

**Table 7:** ANOVA Results of biodiesel yield with KRAM as a catalyst.

Source	Sum of Squares	Df	Mean Square	F-value	p-value	
**Model**	95.74	14	6.84	60.72	<0.0001	Significant
A-Time	3.76	1	3.76	33.39	<0.0001	
B-Irradiation Power	7.02	1	7.02	62.33	<0.0001	
C-Catalyst	0.8363	1	0.8363	7.43	0.0157	
D-MeOH:oil ratio	0.5766	1	0.5766	5.12	0.0389	
AB	23.14	1	23.14	205.44	<0.0001	
AC	1.13	1	1.13	10.07	0.0063	
AD	0.0020	1	0.0020	0.0180	0.8951	
BC	1.82	1	1.82	16.18	0.0011	
BD	0.4900	1	0.4900	4.35	0.0545	
CD	38.50	1	38.50	341.88	<0.0001	
A^2^	5.52	1	5.52	49.05	<0.0001	
B^2^	0.0084	1	0.0084	0.0746	0.7885	
C^2^	13.15	1	13.15	116.80	<0.0001	
D^2^	2.90	1	2.90	25.72	0.0001	
**Residual**	1.69	15	0.1126			
Lack of Fit	0.8320	10	0.0832	0.4852	0.8454	not significant
Pure Error	0.8574	5	0.1715			
**Cor Total**	97.43	29				

The statistical parameters presented in Table 8 were used to assess the model’s fitness. Standard deviation of the analyzed data is close to zero which suggests minimum variability in experimental results. Likewise, experimental results were consistent as shown by the proximity of the R^2^, Adjusted R^2^, and Predicted R^2^. Further supporting the validity of the result is the R^2^ of 0.9827, which demonstrates that the model can account for 98.27% of the variability found in the model response. The Adjusted R^2^ and Predicted R^2^ are reasonably in agreement, with the difference between these parameters being less than 0.2, indicating that there is a strong correlation between the predicted values and the experimental values obtained for WMSO transesterification. Additionally, an adequate precision of 29.4005 indicates that the model has sufficient signals to navigate the design space, a value greater than 4 is desirable, and hence the model can be used for the optimization of WMSO transesterification.

**Table 8:** Model fitness parameters.

Parameters	Values
Standard deviation	0.3356
Mean	89.81
C.V. %	0.3737
R^2^	0.9827
Adjusted R^2^	0.9665
Predicted R^2^	0.9381
Adequate Precision	29.4005

**Fig. 5:**
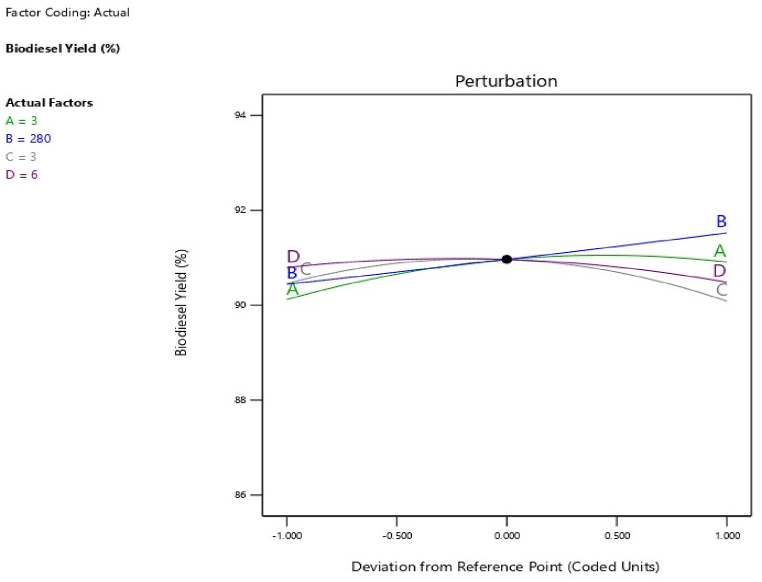
Perturbation plot of biodiesel yield against deviation.

**Fig. 6:**
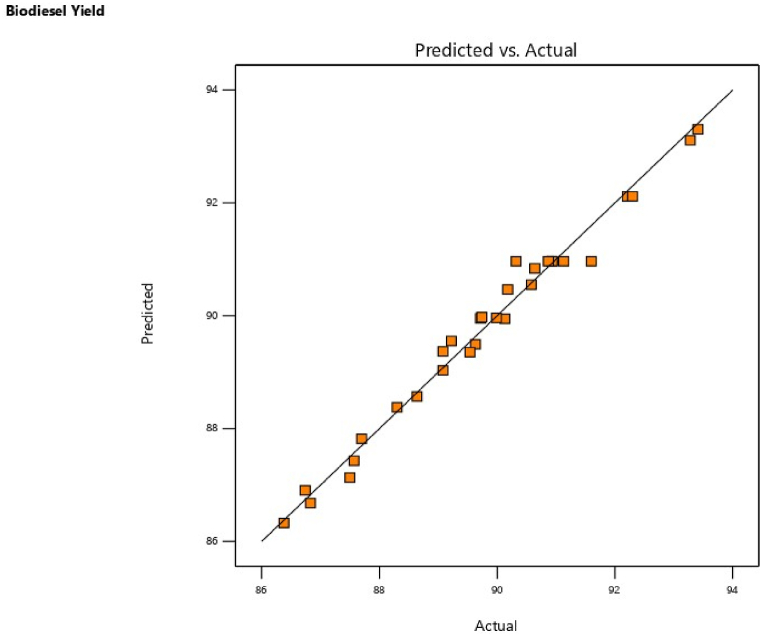
Plot predicted biodiesel yield against actual biodiesel yield.

The regression equation which relates the mathematical relationship between the model response and the four independent variables is expressed in Equation (4) in terms of coded variables.

(4) $$BY\;\left(wt.\%\right)=+108.56167-2.55417A-0.052774B-2.83417C-1.09625D+0.017179AB+0.266250AC+0.005625D+0.004821BC-0.001250BD+0.775625CD-0.448750A^{2}+0.00000357143B^{2}-0.692500C^{2}-0.081250D^{2}$$

where, *BY* (biodiesel yield) is the response, +108.56167 (intercept term), and 2.55417, 0.052774, 2.83417 and 1.09625 are linear term coefficients. The independent variables A, B, C, and D represent time, irradiation power, catalyst amount, and MeOH/WMSO molar ratio, respectively. The degree of influence of each independent variable on the biodiesel yield is more positive than negative. The perturbation plot (Fig. 5) shows the model response against the parameter deviation, indicating the relative degree to which the variables influence biodiesel yield in an ideal situation and away from the referenced point. It can be seen that B and A had a higher deviation than D and C, this observation is consistent with the ANOVA results. Also, the point of intercept is the optimum yield (91.6 wt%) obtained from actual/input factors of 3 min, 280 W, 3 wt% of catalyst, and MeOH/oil ratio of 6:1 corroborated by run 3 in Table 6. Moreover, the integrity of the data obtained and the chosen model are further understood by the plot of predicted biodiesel yield against actual biodiesel yield (Fig. 6). All the data points nearly aligned on the line of fitness, showing a good approximation of the model. This further corroborates the model’s high coefficient of correlation obtained in the statistical parameter of the ANOVA.

**Fig 7:**
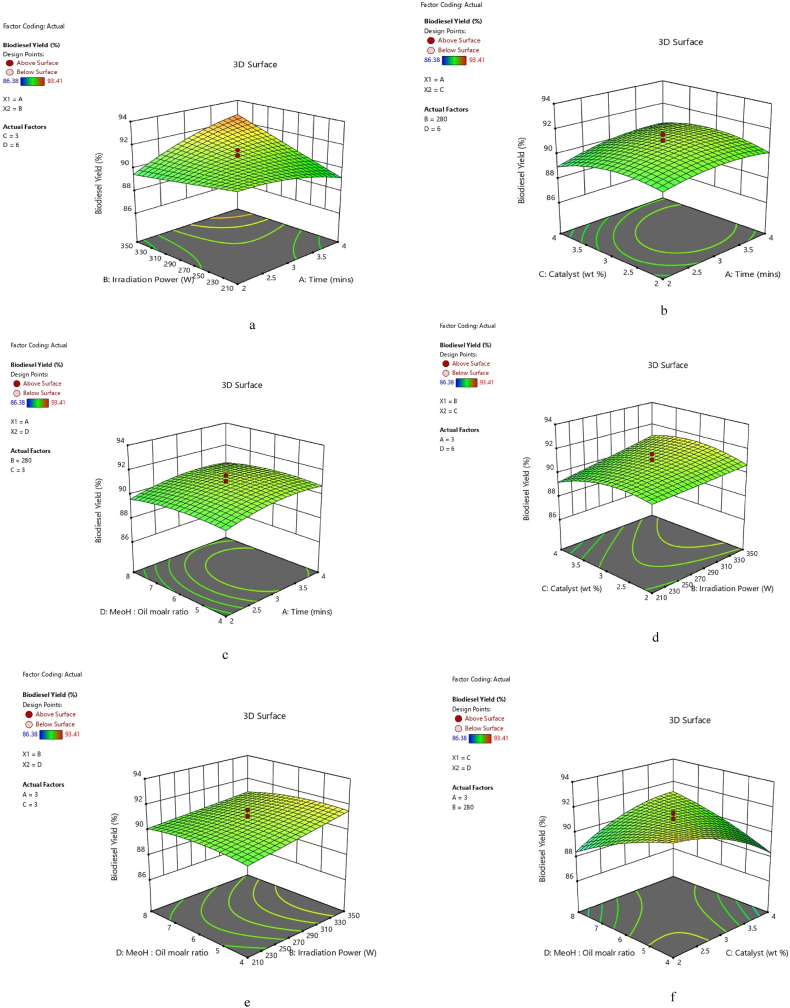
a: 3D time and irradiation power effect. b: 3D time and catalyst effect. c: 3D Time and MeOH/oil molar Effect. d: 3D irradiation power and catalyst effect. e: 3D Irradiation Power and MeOH/oil molar Effect. f: 3D Catalyst and MeOH/oil molar Effect.

Note: Red colour denotes the highest yield, while yellow colour denotes a decent yield that is just slightly above average. Blue colour indicates low output, whereas green colour indicates average yield.

The 3D surface plots generated from the regression equation are represented in Figs. 7a–f. The interaction between variables within the region considered and the optimum biodiesel yield is graphically explored using three-dimensional surfaces. In Fig. 7a, biodiesel yield increases with an increase in time and irradiation power. The increase in both irradiation power and time resulted in increased biodiesel yield. High microwave energy combined with increased time drives the transesterification in a forward direction. High biodiesel production was boosted by microwave-assisted transesterification’s fast reaction time [6]. This is corroborated by the red colour which is a sign of the highest biodiesel yield. Biodiesel production as a result of catalyst and time was average, as indicated by the green colour in Fig. 7b. The production of biodiesel does, however, only slightly rise as time and catalyst amounts are increased. In Fig. 7c, an increase in time and MeOH/oil molar ratio leads to a slight increase in biodiesel yield. And the green colour symbolizes the optimum biodiesel yield. In Fig. 7d, an increase in irradiation power and catalyst amount leads to an increase in biodiesel yield, which is better than in Figures b and c. In Fig. 7e, an increase in irradiation power and a decrease in MeOH/oil molar leads to an increase in biodiesel yield. This is corroborated by the yellow colour, which is a higher yield than green colour. Fig. 7f shows that biodiesel yield increases with decreasing catalyst amount and MeOH/oil molar. Thus, the interaction of both time and irradiation power has a positive effect on biodiesel yield as it increased with an increase in both variables.

### 3.4 Predictive results from python coding

The machine learning techniques could monitor and control biodiesel systems in real-time to enhance production efficiency [42] superior to traditional modeling. Results obtained for biodiesel yield within the data considered for each variable from the python coding algorithms are presented in Table 9. The Python language developed to obtain biodiesel yield using the process variables (time, irradiation power, catalyst amount, and MeOH/oil molar ratio) is presented in the appendix. The predicted biodiesel yields from machine learning are compared to the actual biodiesel yields and the predicted values from the CCD model. The comparison of which modeling tool indicates that the linear regression model technique of machine learning gives a better result as compared to CCD rotatable. Thus, machine learning generates better statistical values and visualization plots mathematically than CCD. Although the linear regression model technique of ML is superior to CCD in modeling and optimizing biodiesel yield, both can accurately forecast the dynamics of biodiesel generation using microwave-assisted transesterification methods. The extra information (Appendix) contains the ML used in this study, which can be recreated by the user’s actual demand.

**Table 9:** Process variables and biodiesel yield from Linear Regression Model of ML.

Run	Time (mins)	Power (W)	Catalyst (wt.%)	MeOH:oilratio	Yield (wt.%)
**0**	3	280	3	10	89.354146
**1**	2	350	4	8	89.551725
**2**	3	280	3	6	90.964146
**3**	3	280	3	2	89.974146
**4**	4	350	2	8	89.367325
**5**	4	350	4	8	93.303685
**6**	3	140	3	6	89.953094
**7**	4	350	4	4	90.838685
**8**	5	280	3	6	89.961046
**9**	3	280	3	6	90.964146
**10**	2	210	2	8	89.029509
**11**	2	350	2	4	90.465365
**12**	4	210	4	4	86.327829
**13**	3	420	3	6	92.114806
**14**	1	280	3	6	88.377246
**15**	4	350	2	4	93.107325
**16**	2	210	4	8	90.550989
**17**	3	280	3	6	90.964146
**18**	2	210	4	4	87.430989
**19**	3	280	5	6	87.820566
**20**	2	350	2	8	86.680365
**21**	3	280	3	6	90.964146
**22**	2	350	4	4	87.131725
**23**	4	210	2	8	86.906349
**24**	3	280	1	6	88.567726
**25**	2	210	2	4	92.114509
**26**	4	210	2	4	89.946349
**27**	3	280	3	6	90.964146
**28**	3	280	3	6	90.964146
**29**	4	210	4	8	89.492829

Average yield = 89.81 wt%.

The model performance for biodiesel yield from WMSO was validated by R^2^, MAE, and RSME. The statistical parameters generated from ML are displayed in Table 10. The chi-squared value and the associated p-value indicate that the biodiesel yield and the variables are statistically significant. The relatively low value of MAE and RSME indicate that the errors are unbiased and it follows a normal distribution. This could mean that the datasets are accurately fitted. The regression model generated as well as the significance of model terms and quadratic terms are presented in Table 12. Similar to the ANOVA results obtained from CCD rotatable modeling, the model terms are all significant. The correlation of the coefficient shows no noticeable variation between the actual and generated biodiesel yields. The R^2^ and Adjusted R^2^ values from the CCD rotatable are similar to the values of the OLS regression model of ML (Table 12). Also, the average yield (89.81 wt%) of the ML is the same as the mean value of the biodiesel yield from CCD rotatable as corroborated in Table 8.

**Table 10:** ML generated statistics.

Parameters	Value
Chi^2^	16745.030139
p-value	0.000001
MAE	9.677592061052564e-13
RMSE	9.837475316895369e-07
Predicted R^2^	1.00

Despite the fact that the ML standard deviation (1.816937) in Table 11 is higher than the CCD rotatable standard deviation (0.3356) in Table 8, the ML approach has more advantages. The 3D plot generated by ML is more visually appealing than the one generated from rotatable CCD. In addition, the values of the OLS regression model of ML are more robust than the ANOVA values generated by CCD rotatable of RSM. Nevertheless, it should be noted that the skewness (−0.425) and kurtosis (2.722) obtained from the OLS Regression of ML are within acceptable boundaries. Although the linear regression model technique of ML is superior to CCD in modeling and optimizing biodiesel yield, both can model biodiesel production with high accuracy.

**Table 11:** Standard deviation of all measurements made.

Time	0.909718
Power	63.680236
Catalyst	0.909718
MeOH/oil molar	1.819435
AB	22.618596
AC	3.930429
AD	7.860859
BC	322.618596
BD	645.237192
CD	7.860859
A_Squared	5.548532
B_Squared	35993.700598
C_Squared	5.548532
D_Squared	22.194128
Yield	1.816937
dtype: float64

**Table 12:** OLS regression result from linear regression model of ML.

Dep. Variable:	Yield	R-squared:	1.000
Model:	OLS	Adj. R-squared:	1.000
Method:	Least Squares	F-statistic:	2.300e+20
Date:	Sat, 04 Feb 2023	Prob (F-statistic):	7.43e-150
Time:	11:50:52	Log-Likelihood:	642.26
No. Observations:	30	AIC:	-1255.
Df Residuals:	15	BIC:	-1233.
Df Model:	14		
Covariance Type:	nonrobust		

Intercept	108.5617	1.4e-09	7.76e+10	0.000	108.562	108.562
Time	-2.5542	3.22e-10	-7.94e+09	0.000	-2.554	-2.554
Power	-0.0528	4.97e-12	-1.06e+10	0.000	-0.053	-0.053
Catalyst	-2.8342	3.22e-10	-8.81e+09	0.000	-2.834	-2.834
MeoH_oil_ratio	-1.0962	1.61e-10	-6.82e+09	0.000	-1.096	-1.096
AB	0.0172	6.16e-13	2.79e+10	0.000	0.017	0.017
AC	0.2663	4.31e-11	6.18e+09	0.000	0.266	0.266
AD	0.0056	2.16e-11	2.61e+08	0.000	0.006	0.006
BC	0.0048	6.16e-13	7.83e+09	0.000	0.005	0.005
BD	-0.0013	3.08e-13	-4.06e+09	0.000	-0.001	-0.001
CD	0.7756	2.16e-11	3.6e+10	0.000	0.776	0.776
A_Squared	-0.4488	3.29e-11	-1.36e+10	0.000	-0.449	-0.449
B_Squared	3.561e-06	6.72e-15	5.3e+08	0.000	3.56e-06	3.56e-06
C_Squared	-0.6925	3.29e-11	-2.1e+10	0.000	-0.693	-0.692
D_Squared	-0.0813	8.23e-12	-9.87e+09	0.000	-0.081	-0.081

According to the results, the Kurtosis score is 2.722, which is considered platykurtic and is favorable because it falls within the permissible range of −3 to +3. Kurtosis can be defined as a measurement of the probability distribution or the width of a distribution’s tails. If a normal distribution’s kurtosis is 3, it is said to be mesokurtic. If it is more than 3, it is referred to as platykurtic, and if it is less than 3, it is referred to as leptokurtic kurtosis. The skew is used to describe how much a distribution leans left or right. It is a distribution’s third instant. The distribution is roughly symmetrical if the skewness is between −0.5 and+0.5 [43]. A curve with a negative skew tends to the right. As can be seen, our skew value is −0.425, which indicates that the distribution is symmetrical, and this is a desirable outcome.

**Fig. 8:**
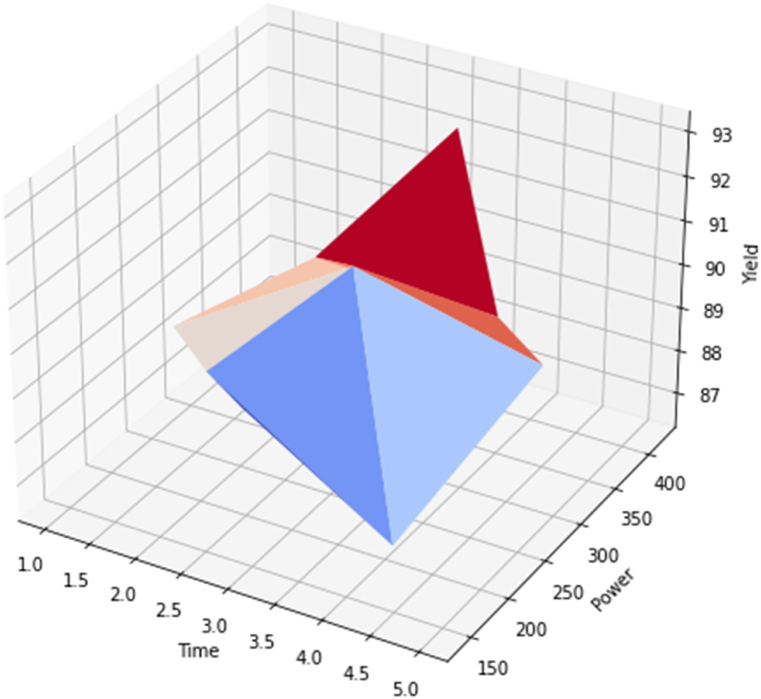
3D effect of time and irradiation power of the biodiesel yield from ML

From Fig. 8, the main actual/input factors considered are Time and Irradiation power while the response/output is the yield. Each colour of a triangle represents an increase in its factor as it moves toward the tip. Sky-blue represents power, blue represents time, milky white represents catalyst, light-peach represents MeOH/oil ratio, brown represents average yield, and red represents optimum yield. However, the intercept of the four input factors and the yield gave the average value which is 89.81 wt% as corroborated in Table 9. And the red colour indicates the optimum value is 91.7 wt% biodiesel yield.

### 3.5 Optimization and validation of experimental data

The optimization for the process input variables was investigated by solving the regression equation. The optimum values predicted were a time of 3 min, irradiation power of 280 W, catalyst amount of 3 wt%, and MeOH/oil molar ratio of 6:1, with a predicted biodiesel yield of 91.60 wt% at the desirability of 1.0. The optimal condition of the input variables was used to carry out the transesterification of WMSO to validate the predicted model. The validation experiment at the optimal condition was conducted three times, and an average of 91.60 wt% biodiesel yield was obtained. The approximation value of biodiesel yield indicates that the model can accurately represent the biodiesel response.

### 3.6 Results of FT-IR analysis of biodiesel produced

**Fig. 9:**
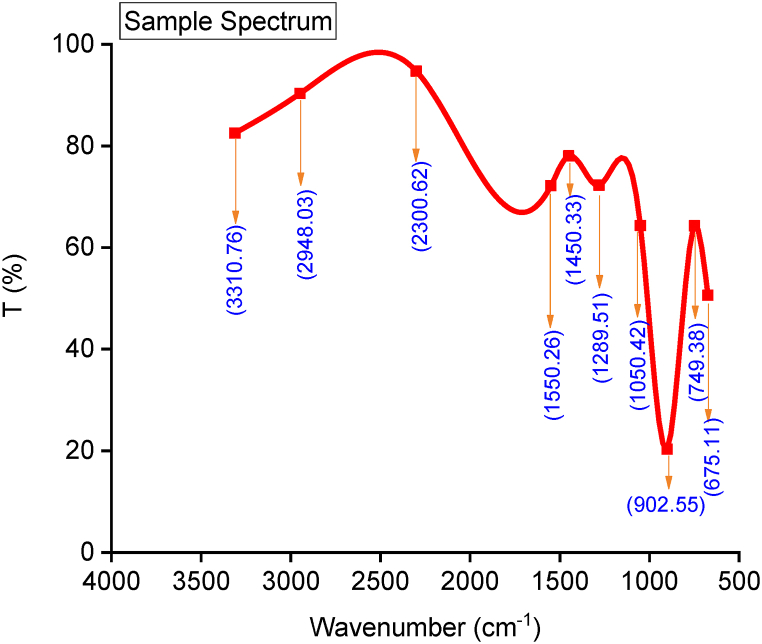
FTIR Spectra of Biodiesel produced.

A Fourier transform infrared spectrometer (Infrared spectrometer Varian 660 MidIR Dual MCT/DTGS Bundle with ATR) identifies the functional groups present in the synthesized biodiesel. Fig. 9 shows the FTIR spectrum and band characteristics of the methyl ester synthesized replotted with Origin Software. The bands at 3310, 2300, and 1550 cm^−1^ are overtone, fermi resonance, and stretching vibrations of the ester functional group, respectively [6]. The CH_2_ group’s asymmetric/symmetric stretching is attributed to the band at 2948 cm^−1^. Between 1550 cm^−1^ and 675.11 cm^−1^ bands is the fingerprint region identifying biodiesel. The stretching, and bending vibration of C–O and CH_2_ groups is assigned to 1289.59 cm^−1^. According to Ref. [44], the band at 1050.42 cm^−1^ indicates the presence of oxygen, suggesting that the biodiesel produced would lead to complete combustion when used in a diesel engine. The bands present in the spectrum of the synthesized biodiesel are common to previously reported bands [6,45].

### 3.7 Characterization of biodiesel produced with GCMS

The methyl esters contained in the synthesized biodiesel from WMSO are displayed in Table 13. The saturated, unsaturated, and polyunsaturated fatty acid methyl acids are present in different proportions.

**Table 13:** GCMS analysis of watermelon seed biodiesel.

Peak	RT	Compound Detected	Mol. Formula	Type of Hydrocarbon
1	8.50	8,11-Octadecadienoic acid, methyl ester	C_19_H_34_O_2_	Unsaturated FAME
2	9.52	9-Hexadecenoic acid, methyl ester, (Z)	C_17_H_32_O_2_	Unsaturated FAME
3	15.15	Dodecanoic acid, methyl ester	C_13_H_26_O_2_	Saturated FAME.
4	16.00	Pentadecanoic acid,13-methyl ester	C_16_H_32_O_2_	Saturated FAME.
5	21.00	Methyl tetradecanoate	C_15_H_30_O_2_	Saturated FAME.
6	25.00	9,12,15-Octadecatrienoic acid, methyl ester	C_19_H_32_O_2_	Polyunsaturated FAME
7	32.00	9-Octadecenoic acid, methyl ester	C_19_H_36_O_2_	Unsaturated FAME
8	34.50	Hexadecanoic acid, methyl ester	C_17_H_34_O_2_	Saturated FAME
9	35.81	Tridecanoic acid, methyl ester	C_14_H_28_O_2_	Saturated FAME
10	37.52	Docosanoic acid, methyl ester	C_23_H_46_O_2_	Saturated FAME
11	39.00	Eicosanoic acid, methyl ester	C_21_H_42_O_2_	Saturated FAME
12	41.98	Methyl stearate	C_19_H_38_O_2_	Saturated FAME
13	44.50	Tetracosanoic acid, methyl ester	C_25_H_50_O_2_	Saturated FAME

### 3.8 Physicochemical properties of biodiesel produced

The synthesized biodiesel’s physicochemical properties were identified and evaluated against standards set by the European Union, the American Society for Testing and Materials, *Acacia Farnesiana* oil biodiesel (AFOB) and *Albizzia julibrissin* oil biodiesel (AJOB). The synthesized biodiesel’s (WMSO biodiesel) in this study were determined in triplicate. It can be seen that the synthesized biodiesel met the specifications stipulated by the EU and ASTM standards. However, Table 14 shows that the biodiesel produced performs better than *Acacia Farnesiana* oil biodiesel and *Albizzia julibrissin* oil biodiesel in terms of flash point, cloud point, kinematics viscosity, cetane number, etc. As a result, the synthesized biodiesel could be used in the internal combustion engine without any modification.

**Table 14:** Physicochemical properties of Biodiesel produced.

Properties	This Study	EN 14214	ASTM D6751	AFOB^5^	AJOB^7^
Density (kg/m^3^)	871.7	860–900	880	831	842.44
Specific gravity	0.8717	NS	NS	0.83134	0.84244
Acid value (mg KOH/g)	0.34	0.5 max	0.5 max	0.4	0.2
Flash point (°C)	132	<120	100–170	158	160
Cloud point (°C)	−3	NS	−3 to 12°C	7	9
Pour point (°C)	−10	NS	−15 to 16	−28	−12
Kinematic viscosity at 40 °C (mm^2^/s)	2.16	3.5–5	1.9–6	5.32	3.75
Cetane number	63.95	51 min	47 min	52	58
Fire point (°C)	150	NS	NS	189	190
Refractive index	1.51	NS	NS	NS	NS
Saponification value (mgKOH/g)	172	NS	NS	174.8	180.4
Iodine value (g I_2_/100 g oil)	1.84 Ig/100 g	<120	NS	142.5	118.5
Calorific value (MJ/Kg)	85.93	35	NS	NS	NS

NS Not supplied.

References for comparative analysis: **AFOB**^**5**^**:***Acacia Farnesiana* oil biodiesel, AJOB^7^:*Albizzia julibrissin* oil biodiesel.

## 4 Conclusion

A heterogeneous base catalyst of high catalytic efficiency was synthesized from the calcination of Kwale red anthill mud (KRAM). The synthesized catalyst in this study was predominantly Si and Al oxides or compounds, these are believed to be responsible for the catalytic capability of the solid basic catalyst. Additionally, high porosity and possession of a large surface area (42.16 m^2^/g) on the synthesized catalyst signal that reacting species in the transesterification could access its active site unhindered, thereby facilitating biodiesel production. The oil used for biodiesel production was extracted from dried watermelon seeds via the Soxhlet extraction principle using *n*-hexane as solvent. Two modeling tools; Central Composite Design (CCD) rotatable and (Machine learning) python coding was successfully used to model and optimize biodiesel production from WMSO and methanol in the presence of a synthesized catalyst. Python coding generated a model with better performance than CCD rotatable. The model R^2^ obtained using CCD rotatable was 0.9827 while the R^2^ of the linear regression model of ML was 1, and the 3D graph generated by ML was more visually appealing. ML outperforms CCD rotatable of RSM in forecasting and optimizing biodiesel output, and it also provides additional statistical factors that could help with decision-making. The highest yield in this study is at 4 wt% catalyst amount, 4 min duration, methanol/oil molar 8:1, and irradiation power of 350 W with the corresponding biodiesel yield of 93.41 wt%. The biodiesel produced in this study was within the allowable limit specified by the standards. The catalyst synthesized proved to be a good heterogeneous catalyst capable of expediting biodiesel production from watermelon seed oil.

## Declarations research funding

This study has not received any financial assistance.

## Author contribution statement

Sunday Chukwuka Iweka: Conceived and designed the experiments; Performed the experiments; Analyzed and interpreted the data; Contributed reagents, materials, analysis tools or data; Wrote the paper.

Olayomi Abiodun Falowo; Amosun Adebimpe Amos; Eriola Betiku: Analyzed and interpreted the data.

## Data availability statement

Data will be made available on request.

## Declaration of competing interest

The authors declare that they have no known competing financial interests or personal relationships that could have appeared to influence the work reported in this paper

## Figures and Tables

**Table:** 

**Python Coding (ML)**
In [1]: Import numpy as np
import seaborn as sns
from matplotlib import cm
from matplotlib.ticker import LinearLocator, FormatStrFormatter
from sklearn.metrics import mean_absolute_error, mean_squared_error, r2_score, mean_absolute_percentage_error
from mpl_toolkits.mplot3d import Axes3D
from scipy.interpolate import griddata
In [2]: data = {"Time":[3, 2, 3, 3, 4, 4, 3, 4, 5, 3, 2, 2, 4, 3, 1, 4, 2, 3, 2, 3, 2, 3, 2, 4, 3, 2, 4, 3, 3, 4],
“Power":[280, 350, 280, 280, 350, 350, 140, 350, 280, 280, 210, 350, 210, 420, 280, 350, 210, 280, 210, 280, 350, 280, 350, 210, 280, 210, 210, 280, 280, 210],
“Catalyst":[3, 4, 3, 3, 2, 4, 3, 4, 3, 3, 2, 2, 4, 3, 3, 2, 4, 3, 4, 5, 2, 3, 4, 2, 1, 2, 2, 3, 3, 4],
“MeoH_oil_ratio":[10, 8, 6, 2, 8, 8, 6, 4, 6, 6, 8, 4, 4, 6, 6, 4, 8, 6, 4, 6, 8, 6, 4, 8, 6, 4, 4, 6, 6, 8] }
In [3]: df = pd.DataFrame(data)
In [4]: a = df['Time']
b = df['Power']
c = df['Catalyst']
d = df['MeoH_oil_ratio']
df["AB"] = a*b
df["AC"] = a*c
df["AD"] = a*d
df["BC"] = b*c
df["BD"] = b*d
df["CD"] = c*d
df["A_Squared"] = a**2
df["B_Squared"] = b**2
df["C_Squared"] = c**2
df["D_Squared"] = d**2
df
In [5]: df['Yield'] = 108.56167–2.55417*a - 0.052774*b - 2.83417*c - 1.09625*d + 0.017179*a*b + 0.266250*a*c + 0.005625*a*d + 0.004821*b*c - 0.001250*b*d + 0.775625*c*d - 0.448750*a**2 + 0.00000356143*b**2–0.692500*c**2–0.081250*d**2
df
In [6]: df2 = df.iloc[:,0:4]
df3 = df.iloc[:,-1]
df4 = pd.concat([df2,df3],axis = 1)
df4
In [7]: yield_average = round(df['Yield'].mean(), 2)
yield_average
In [8]: df.columns
In [9]: df.cov()
In [10]: df.corr()
In [11]: df.describe()
In [12]: chi2, pval, dof, expected = stats.chi2_contingency(df)
print(“Statistics: ")
print(“--------------")
print(“Chi2 = %f, p-value = %f"%(chi2, pval))
In [13]: #X = df.drop(‘Yield’, axis = 1)
#y = df['Yield']
#model = smf.ols(“y ∼ X″, df).fit()
model = smf.ols(“Yield ∼ Time + Power + Catalyst + MeoH_oil_ratio + AB + AC + AD + BC + BD + CD + A_Squared + B_Squared + C_Squared + D_Squared”, df).fit()
model
In [14]: print(model.summary())
In [15]: from sklearn.linear_model import LinearRegression
lr = LinearRegression()
lr.fit(X_train, y_train)
In [16]: lr.score(X_test, y_test)
In [17]: pred = lr.predict(X_test)
print(“predicted values are:",pred)
In [18]: mae = mean_absolute_error(y_test, pred)
print(‘mae = ', mae)
rmse = mae**0.5
print(‘rmse = ', rmse)
predicted_R_squared = r2_score(y_test, pred)
print(“predicted_R_squared = ", round(predicted_R_squared, 5))
In [19]: from mpl_toolkits.mplot3d import Axes3D
import matplotlib.pyplot as plt
import random
from matplotlib import cm
fig = plt.figure(figsize=(10,8))
ax = fig.add_subplot(111, projection = ‘3d’)
x = df[‘Time’]
y = df[‘Power’]
z = df[‘Yield’]
surf = ax.plot_trisurf(x, y, z, cmap = cm.coolwarm, linewidth = 0.2)
ax.set_xlabel(‘Time’)
ax.set_ylabel(‘Power’)
ax.set_zlabel(‘Yield’)
In [20]: std2 = df.std()
print(std2)
In [21]: df.to_csv(“./MrChukwuka.csv”, index = False)
